# Antiproliferative effect of Tualang honey on oral squamous cell carcinoma and osteosarcoma cell lines

**DOI:** 10.1186/1472-6882-10-49

**Published:** 2010-09-14

**Authors:** Abdulmlik A Ghashm, Nor H Othman, Mohammed N Khattak, Noorliza M Ismail, Rajan Saini

**Affiliations:** 1 School of Dental Sciences, Universiti Sains Malaysia, 16150 Kubang Kerian, Kelantan, Malaysia; 2 School of Medical Sciences, Universiti Sains Malaysia, 16150 Kubang Kerian, Kelantan, Malaysia

## Abstract

**Background:**

The treatment of oral squamous cell carcinomas (OSCC) and human osteosarcoma (HOS) includes surgery and/or radiotherapy which often lead to reduced quality of life. This study was aimed to study the antiproliferative activity of local honey (Tualang) on OSCC and HOS cell lines.

**Methods:**

Several concentrations of Tualang honey (1% - 20%) were applied on OSCC and HOS cell lines for 3, 6, 12, 24, 48 and 72 hours. Morphological characteristics were observed under light and fluorescent microscope. Cell viability was assessed using MTT assay and the optical density for absorbance values in each experiment was measured at 570 nm by an ELISA reader. Detection of cellular apoptosis was done using the Annexin V-FITC Apoptosis Detection Kit.

**Results:**

Morphological appearance showed apoptotic cellular changes like becoming rounded, reduction in cell number, blebbed membrane and apoptotic nuclear changes like nuclear shrinkage, chromatin condensation and fragmented nucleus on OSCC and HOS cell lines. Cell viability assay showed a time and dose-dependent inhibitory effect of honey on both cell lines. The 50% inhibitory concentration (IC_**50**_) for OSCC and HOS cell lines was found to be 4% and 3.5% respectively. The maximum inhibition of cell growth of ≥80% was obtained at 15% for both cell lines. Early apoptosis was evident by flow cytometry where percentage of early apoptotic cells increased in dose and time dependent manner.

**Conclusion:**

Tualang honey showed antiproliferative effect on OSCC and HOS cell lines by inducing early apoptosis.

## Background

Cancer of the oral cavity is eleventh most common malignancy worldwide [1] while in the Indian subcontinent and regions of Southeast Asia, it is the predominant malignancy accounting for up to 40% of all the cancers [2]. This high incidence of oral cancer is primarily attributable to the habit of tobacco, betel quid chewing and alcohol consumption [3].

Oral squamous cell carcinomas (OSCC) are the most common type of oral cancers. Similarly, Human Osteosarcomas (HOS) that arise from the jaw, account for 2.1% of all malignant oral and maxillofacial tumours [4]. The treatment of these types of oral cancers includes surgery and/or radiotherapy, which are often associated with loss of function, disfigurement and reduced quality of life [5]. Recently, advances in chemotherapeutic agents for the treatment of OSCCs have been highlighted but the survival rate of patients has not improved significantly. The development of novel therapeutic agents targeting the malignant behaviour of these cancers is important to improve the prognosis of treatment [6]. The study of molecular mechanisms of chemotherapeutic agents and the combination of chemotherapeutic agents that induce synergistic anticancer activity are necessary to improve clinical outcomes [7].

Many researchers have studied the anticancer activities of drugs or herbal extracts on OSCC cell lines. These include Tamoxifen in combination with Cisplatin [7], 5-Fluorouracil [8], Cordycepin [9], Scutellaria baicalensis [10], Quercetin [11] Artemisinin [12] and others. Similarily, Ginsenoside Rg1, Cinnamic acid, and Tanshinone IIA [13], Diosgenin[14], Venenum Bufonis and Oxgall powder[15] have been shown to have antiproliferative effect on HOS cell lines.

Honey is a food product which is collected from various plants and processed by honey bees (Apis mellifera). Honey has been used as traditional medicine for centuries in different cultures, not only for its nutritional value but also its healing properties. Recently, honey has been tested and approved scientifically for its functional and biological properties like anti-oxidant, anti-inflammatory, anti-bacterial, anti-viral, anti-ulcerous activities, anti-lipid and anti-cancer properties [16-23]. These activities are mainly attributed to the phenolic compounds such as flavonoids having antioxidant properties and radical scavenging activities seen among all types of honeys in different proportions, depending on the geographical areas, source of honeybee food and climate [24-26].

Honey has also been used in palliative care of various cancers like in radiation-induced mucositis, radiotherapy and chemotherapy induced skin reactions and wounds [27]. It has also been shown to produce antiproliferative effects in bladder cancer [21], colon cancer [28], mammary carcinoma and fibrosarcoma [22]. However, till date no study has been found to show antiproliferative effects of honey on oral cancers.

Malaysian Tualang honey is collected from the honey combs of Asian rock bees (*Apis dorsata*), which build their hives high up in the Tualang tree (*Koompassia excelsa*). Tualang honey is used commonly as a medicinal product [29] and as a food in Malaysia. Recently, antibacterial properties of this honey have been studied and compared with other honeys [30,31]. However, its antiproliferative properties are yet to be studied. The purpose of the current study was to investigate the antiproliferative activity of Malaysian Tualang honey on OSCC and HOS cell lines.

## Methods

### Honey

Local Malaysian honey (Tualang honey), which was donated by the Food and Agricultural Ministry (FAMA), was used in this study. Working concentrations of honey were prepared fresh for each experiment by serial dilution with culture medium after which each concentration was filtered using 0.20 μm sterile filter unit (Sartorius stedim).

### Cell culture materials

OSCC cell lines were purchased from American Tissue Collection Centre (ATCC) (CRL-1623) and were maintained in Dulbecco's Modified Eagle's Medium (DMEM) and Ham's F12 (DMEM/F12) (Sigma-Aldrich, USA). HOS cell lines were purchased from ATCC (CRL-1543) and were maintained in DMEM high glucose 1× (Gibco^® ^invitrogen USA). Culture media was supplemented with 10% Foetal Bovine Serum (FBS) and 1% penicillin/streptomycin (Gibco^® ^invitrogen).

### Morphological analysis under light and fluorescent microscopy

For morphological analysis, OSCC and HOS cell lines were seeded in 60-mm dishes at 5 × 10^5 ^cells/ml. The cells were treated with 2% and 10% concentration of Tualang honey for 24, 48 and 72 hours. At the indicated time points, morphological changes were examined and recorded under light microscope (Carl Zeiss, Germany). Apoptosis was also determined morphologically at similar intervals and concentrations after staining the cells with Hoechst 33258 (Sigma-Aldrich) at a concentration of 20 μg/ml in PBS and incubated for 30 minutes. The cells were observed in the dark using Axioplan 2 fluorescent microscope (Carl Zeiss, Germany) at 356 nm.

### MTT cell viability assay

OSCC and HOS cell lines were seeded in 96-well plate (Nunc™, Denmark) containing 1 × 10^4 ^cells with 100 μl serum medium. After the cells reached 70-80% confluence, they were treated with serum free medium containing Tualang honey concentrations from 1% to 20% for 3, 6, 12, 24 and 48 hours for all concentrations. 3-(4,5-Dimethylthiazol-2-yl)-2,5-diphenyl-2*H*-tetrazolium bromide or MTT (Calbiochem, Germany) was added at different time points with the final concentration of 0.5 mg/ml and then incubated for 4 hours at 37°C. The medium was removed and Dimethyl Sulphoxide (DMSO) (Ajax Finechem Pty Ltd, Australia) was added to dissolve the crystals by shaking the plate weakly for 15 minutes in the dark [9]. The optical density (O.D.) of each treatment was measured at 570 nm by an ELISA reader (Sunrise, Tecan). Each experiment was performed in triplicates. Considering control untreated cells (cells without Tualang honey) as having 100% proliferation rate, the proliferation of cancer cells was expressed as the % cytoviability using the following formula: % cytoviability = A570 of treated cells/A570 of control cells × 100% [32].

### Non-peroxidase, acidity and osmolar activity

Non-peroxidase activity of Tualang honey was measured by diluting it to 25% (w/v) by taking 1 ml of honey and adding it to either 1 ml of sterile purified water (total activity) or 1 ml of catalase solution. The 8000 U catalase solution (Sigma, C9322: 2950 units/mg) was used to remove all the hydrogen peroxide present in the honey. The removal of hydrogen peroxide was verified according to the method described elsewhere [33]. Acidity of honey concentrations and culture medium was measured by pH 211 meter (Hanna instruments, USA). Osmolar control solution was prepared by mixing sugars namely 4 g fructose, 3 g glucose, 0.2 g sucrose and 0.8 g maltose mixed for 1 hour at 80°C to make in total 10 ml of the solution with distilled water. The solution was adjusted to be of the same osmolarity as that of honey concentrations by using an osmometer (Gonatec, Germany).

### Cell apoptosis assay by flow cytometry

Cellular apoptosis was determined using the Annexin V-FITC Apoptosis Detection Kit I (Clontech Laboratories Inc, USA) according to the manufacturer's protocol. OSCC and HOS cell lines were cultured at 6 × 10^5 ^cells/ml and seeded in 60 mm dish. The cells were treated with free medium containing various concentrations of Tualang honey for 6, 12 and 24 hour. Cells were harvested by trypsinization, then washed twice with cold PBS and centrifuged at 1000 rpm. About 1 × 10^5 ^- 1 × 10^6 ^cells were then resuspended in 400 μl 1× binding buffer, centrifuged again at 1000 rpm for 5 minutes and then supernatant was removed. Cells were re-suspended in 200 μl 1× binding buffer and transferred to a sterile flow cytometry glass tube. Five μl Annexin V-FITC and 10 μl propidium iodide were added and then incubated in the dark at room temperature. Cells were analyzed by flow cytometer (FACSCalibur, Becton-Dickinson, USA) at 488 nm. The distribution of cells was analyzed using CellQuest™ software (Becton-Dickinson) in the flow cytometer within 1 hour of staining. Data from 10,000 cells was collected for each data file. Apoptotic cells were identified as Annexin V-FITC-positive and P-negative cells.

### Statistical analysis

The data from MTT assay was analyzed by SPSS software version 12.0.1 and the results were expressed as median (IQR) of three independent experiments. As the data was not normally distributed and the assumption for equal variances was not fulfilled, Kruskal-Wallis test was applied. The pairwise comparison was analyzed using Mann-Whitney test and Bonferroni correction was applied. The difference in the median between the different concentrations and time points was considered to be statistically significant if the *p-*value was *<*0.05.

## Results

### Morphological changes in OSCC and HOS cell lines under light microscopy

**Figure 1:**
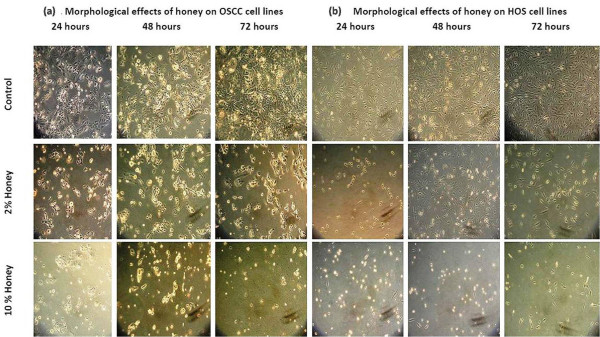
**Effect of Tualang honey on morphology of (a) OSCC and (b) HOS cell lines as seen under light microscope**. Cells were cultured in 6-well plates until 70-80% confluence and then treated with Tualang honey 2% and 10% for 24, 48 and 72 hrs

In both cell lines, cells without Tualang honey treatment showed polygonal shape, which is considered as the normal cell growth phenomenon. However, when the cells were treated with 2% and 10% honey for 24, 48 hours and 72 hours, the cells rounded up and showed reduction in number. Cells with blebbed membrane could also be recognized (Fig. 1). These are the morphological changes typically seen in apoptosis [9,34]. These changes suggested that Tualang honey had induced apoptotic cell death in OSCC and HOS cell lines.

### Morphological changes in OSCC and HOS cell lines under fluorescence microscopy

**Figure 2:**
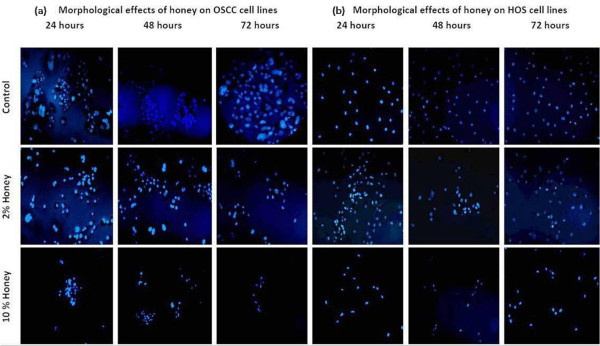
**Morphological changes in nucleus seen in (a) OSCC and (b) HOS cell lines following treatment with 2% and 10% Tualang honey for 24, 48 and 72 hrs**. Cells were stained with Hoechst 33258 and observed under fluorescence microscope.

In honey treated cell lines, morphological alteration such as nuclear shrinkage, chromatin condensation and fragmented nucleus were observed (Fig 2). These morphological changes were characteristic of apoptotic cells.

### Effect of Tualang honey on cell viability

**Figure 3:**
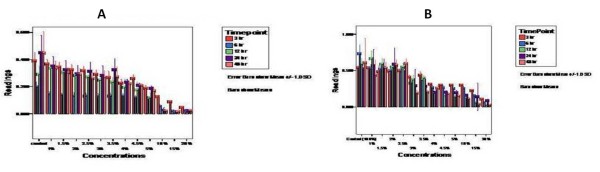
**Graph with error bars showing antiproliferative effect of Tualang honey concentrations on (A) OSCC and (B) HOS cell lines**. Cells were seeded onto 96-well plate at 1 × 10^4^/well and were treated with Tualang honey at different concentrations. Cell viability was determined by MTT assay after 3, 6, 12, 24 and 48 hour of treatment. Time and dose dependent growth inhibition in both cell lines was observed.

The honey concentration required for the 50% inhibition of cell growth (IC50) was calculated after 3, 6, 12, 24 and 48 hours of exposure to honey. After 2 days of culture, the honey IC50 of OSCC cell lines was higher than that of HOS cells, at 4% and 3.5% respectively. These results clarify the time and dose dependent inhibitory effect of Tualang honey on both cell lines. The maximum inhibition of cell growth of ≥80% was obtained at 15% for both OSCC cell lines and HOS cell lines as shown in Fig 3a and Fig 3b respectively.

### Non-peroxidase, acidity and osmolar activity

**Figure 4:**
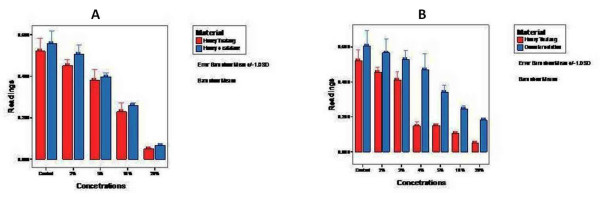
**Graph with error bars comparing the effects of peroxidase activity and osmolality of honey**. (A) MTT assay for non-peroxidase activity of honey. Similar effect as shown by various honey concentrations was seen. (B) Effect of different concentrations of honey on the proliferation rate of HOS cell lines in comparison to analogue osmolar solution controls. Antiproliferative effect of honey was found to be more than its analogue osmolar solution.

MTT assay for non-peroxidase activity of honey showed a similar effect as shown by various honey concentrations, suggesting that this antiproliferative effect was due to various other constituents of honey rather than hydrogen peroxide (Fig. 4A). The effect of acidity of honey was excluded as the pH of blank growth media was found to be similar to the pH of 3.5% honey. The pH of most of the honey concentrations used was within the normal pH range for cell line culture. Antiproliferative effect of honey was found to be more than the osmolar control solution of the same concentration (Fig. 4B).

### Cell apoptosis assay results

**Figure 5:**
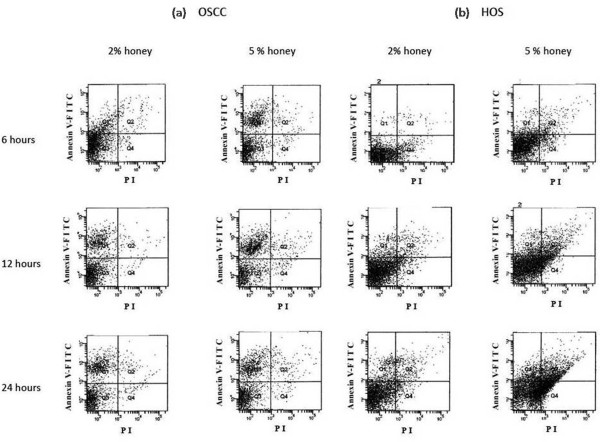
**Apoptotic effects of Tualang honey on (a) OSCC and (b) HOS cell lines were determined by flow cytometry analysis using annexin V-FITC and propidium iodide**. The experiments were performed after treatment with 2% and 5% honey for 6, 12 and 24 hrs. Cell populations in bottom left, bottom right, top right and top left quadrants represented the proportion of viable cells, necrotic cells, late apoptotic cells and early apoptotic respectively.

The dual parameter fluorescent dot plots (Fig 5) shows the viable cell population in quadrant 3 (negative annexin-FITC and negative PI), the cells at the early apoptosis are in quadrant 1 (positive annexin-FITC and negative PI) while the ones at the late apoptosis are in quadrant 2 (positive annexin-FITC and positive PI) [35]. As seen in fig 5, control untreated cells were mostly alive whereas when the honey treatment was applied, the early apoptotic cells percentage increased in relation to its concentration. The box plot percentages of cell populations revealed that effect was dose and time dependent.

## Discussion

Honey, a part of traditional medicine, has recently become the focus of attention for treating certain diseases as well as promoting overall health and well being. Several honey types from different floral sources and geographical regions have been reported to contain many phenolic compounds, which act as antioxidants and exhibit anti-carcinogenic, anti-inflammatory, anti-microbial, anti-atherogenic, anti-thrombotic, immune modulating and analgesic activities [36,37]. In the oral health setting, honey has been found to be effective for the treatment of radiation-induced oral mucositis [38], stomatitis [39], reducing plaque and periodontal gum disease [40] and is also found to be anticariogenic [41].

In this study, we investigated the antiproliferative and apoptotic activities of Malaysian Tualang honey on human OSCC and HOS cell lines. This honey was chosen for the study as antiproliferative effects of this type of honey have not yet been verified on any type of the cancer cell lines. Additionally, in pure unprocessed honey like Tualang honey, there are a number of volatile compounds reported that may be missed in processing and fractionation [21]. This is the first study to report the antiproliferative activity of honey on OSCC and HOS cell lines.

While most of the previous studies on honey have focused on its anti-microbial and wound healing properties, only few papers have looked into the anticarcinogenic properties of honey. Gribel and Pashinskii (1990) reported that honey revealed moderate antitumor and pronounced antimetastatic effects. Honey was also seen to potentiate the antitumor activity of 5-fluorouracil and cyclophosphamide [42]. Wang et al. studied the anti-mutagenic effects of different types of honey against a commonly encountered dietary mutagen Trp-p-1 and found that all honeys exhibited significant inhibition of mutagenicity against this compound [20]. In another study, bee honey was found to be an effective agent for inhibiting the growth of bladder cancer cell lines *in vitro*, and bladder cancer implanted mice models *in vivo *[21]. A study done on tumour development and metastasis in murine tumour models using various honey-bee products showed an important role in controlling tumour growth and metastasis in mammary carcinoma and a methylcholanthrene-induced fibrosarcoma in mouse [22].

The present study showed that unfractionated Tualang honey has potential time and dose dependent antiproliferative effect on OSCC and HOS cancer cell lines. We found that IC_50 _for Tualang honey was 4% for OSCC and 3.5% for HOS cell lines. Non-peroxidase activity of honey was found to show the same effect as shown by various honey concentrations. Further, results of confirmatory experiments ruled out the low pH effect of honey as the reason for its antiproliferative activity. Similarly, the inhibitory effect of honey was found to be more than the effect of analogous osmolar solutions. Thus, hydrogen peroxide activity, acidity and hyperosmolarity of honey were ruled out for its inhibitory effect on these cancer cell lines. A recent article suggested that the polyphenols found in honey, like Caffeic acid, Caffeic acid phenyl esters, Chrysin, Galangin, Quercetin, Kaempferol, Acacetin, Pinocembrin, Pinobanksin and Apigenin, to be promising pharmacological agents in the treatment of cancer by reviewing their antiproliferative and molecular mechanisms [43]. These compounds have been shown to exhibit an inhibitory effect on tumour cell proliferation and transformation by the down regulation of many cellular enzymatic pathways including protein tyrosine kinase, cycloxygenase and ornithine decarboxylase pathways [44]. Another study done on jungle honey obtained from the tropical forest of Nigeria showed that this type of honey possessed chemotactic activity for neutrophils, which were found to possess potent antitumour activity mediated by reactive oxygen species (ROS) [45].

As shown by our flow cytometry results, when the concentration of honey was increased, the percentage of early apoptotic cells also increased. For this reason, the mode of cell death appears to be due to early apoptosis cell death pathway. These apopototic changes were also visible in the morphological studies which were done using light and fluorescent microscopy where membrane blebs, chromatin and nuclear condensation, DNA fragmentation and formation of apoptotic bodies were seen. A change in the status of the intracellular non-protein thiol, fall in MMP and increased ROS generation were reported to occur during apoptosis [46]. Moreover, the expression of various proapoptotic and antiapoptotic proteins was found to be altered during apoptosis [47]. A recent study done to understand the molecular mechanism of honey in colon cancer cell growth inhibition found that honey induced apoptosis was accompanied by up-regulating the p53 and modulating the expression of pro and anti-apoptotic proteins [48]. They also reported that unfractionated honey induced cell-growth arrest, resulting in cell cycle blockage at the sub-G1 phase. Further, it transduced the apoptotic signal via initial depletion of intracellular non-protein thiols (GSH), consequently reducing the MMP and increasing the ROS generation. Similar studies need to be done in OSCC and HOS cell lines to confirm these findings.

## Conclusion

In conclusion, the results of this study suggest that Tualang honey has a promising antiproliferative and apoptotic effect on OSCC and HOS cell lines. Early apoptosis could be attributed, in part, to its ability to inhibit proliferation. Further investigations are needed to determine the molecular mechanisms involved in apoptosis.

## Competing interests

The authors declare that they have no competing interests.

## Authors' contributions

RS designed the research project and drafted the manuscript. AAG carried out the cell culture, microscopy and lab work. MNK calculated the sample size and did the statistical analysis of the study. RS and NHO guided the bench work of the procedure. RS wrote the final manuscript and NHO, NMI critically reviewed it. All authors read and approved the final manuscript.

## Pre-publication history

The pre-publication history for this paper can be accessed here:

http://www.biomedcentral.com/1472-6882/10/49/prepub
